# Non-surgical Management of Inflammatory Anterior Chamber Hypopyon Post Vitreoretinal Surgery for Proliferative Diabetic Retinopathy

**DOI:** 10.7759/cureus.80859

**Published:** 2025-03-19

**Authors:** Akash Belenje, Padmaja K Rani, Vivek P Dave

**Affiliations:** 1 Srimati Kanuri Santhamma Center for Vitreo-Retinal Diseases, Anant Bajaj Retina Institute, Kallam Anji Reddy Campus, L V Prasad Eye Institute, Hyderabad, IND

**Keywords:** hypopyon, postoperative inflammation, post vitreoretinal surgery, proliferative diabetic retinopathy (pdr), type 1 and type 2 diabetes mellitus

## Abstract

We report two unusual cases of inflammatory anterior chamber hypopyon post vitreoretinal surgery for vitreous hemorrhage secondary to proliferative diabetic retinopathy (PDR). Two out of eight patients operated for proliferative diabetic retinopathy developed hypopyon in the anterior chamber one week post-operative. Case number 2 also had an exudative plaque on the iris and a pupillary membrane. No anterior chamber (AC) entry was made during the primary surgery, and none of the other patients who operated on the same day had a similar reaction to any of the intraocular solutions with the same batch numbers used during the surgery. Physician fitness was taken before surgery, and the diabetic profile of all the patients showed a Hemoglobin A1C of less than 8% and random blood sugar of less than 200 mg/dl. The hypopyon resolved completely with frequent topical steroids and cycloplegics without any cover of topical or systemic antibiotics, suggesting it to be an inflammatory response rather than endophthalmitis. Moreover, neither patient had lid edema and tenderness, which are the hallmark presenting features of acute post-surgical endophthalmitis. We attribute this clinical presentation primarily to the exaggerated inflammatory response to surgery in diabetic retinopathy patients. Since diabetic retinopathy is a condition with raised inflammatory markers, these patients are more prone to an exaggerated inflammatory response to an event like vitreoretinal surgery where various blood ocular barriers could be compromised.

## Introduction

Several studies have shown the upregulation of inflammatory markers in tears, aqueous, and vitreous in patients with diabetic retinopathy [1,2]. These inflammatory markers are known to disrupt various blood-ocular barriers, resulting in faster recruitment of inflammatory mediators and cells [3,4]. Pro-inflammatory cytokines and chemokines, like interleukin (IL-1ß, IL-6) interferon-γ (INF-γ), tumor necrosis factor-α (TNF-α), and intracellular adhesion molecules (ICAM) are the raised inflammatory markers in diabetic retinopathy patients [2,3]. When retinopathy changes are severe, especially PDR, the expression of these inflammatory markers is higher [4]. An acute event like vitreoretinal surgery can be a triggering factor for inflammatory hypopyon in diabetic retinopathy patients, and we describe two such cases.

## Case presentation

Case 1

**Figure 1:**
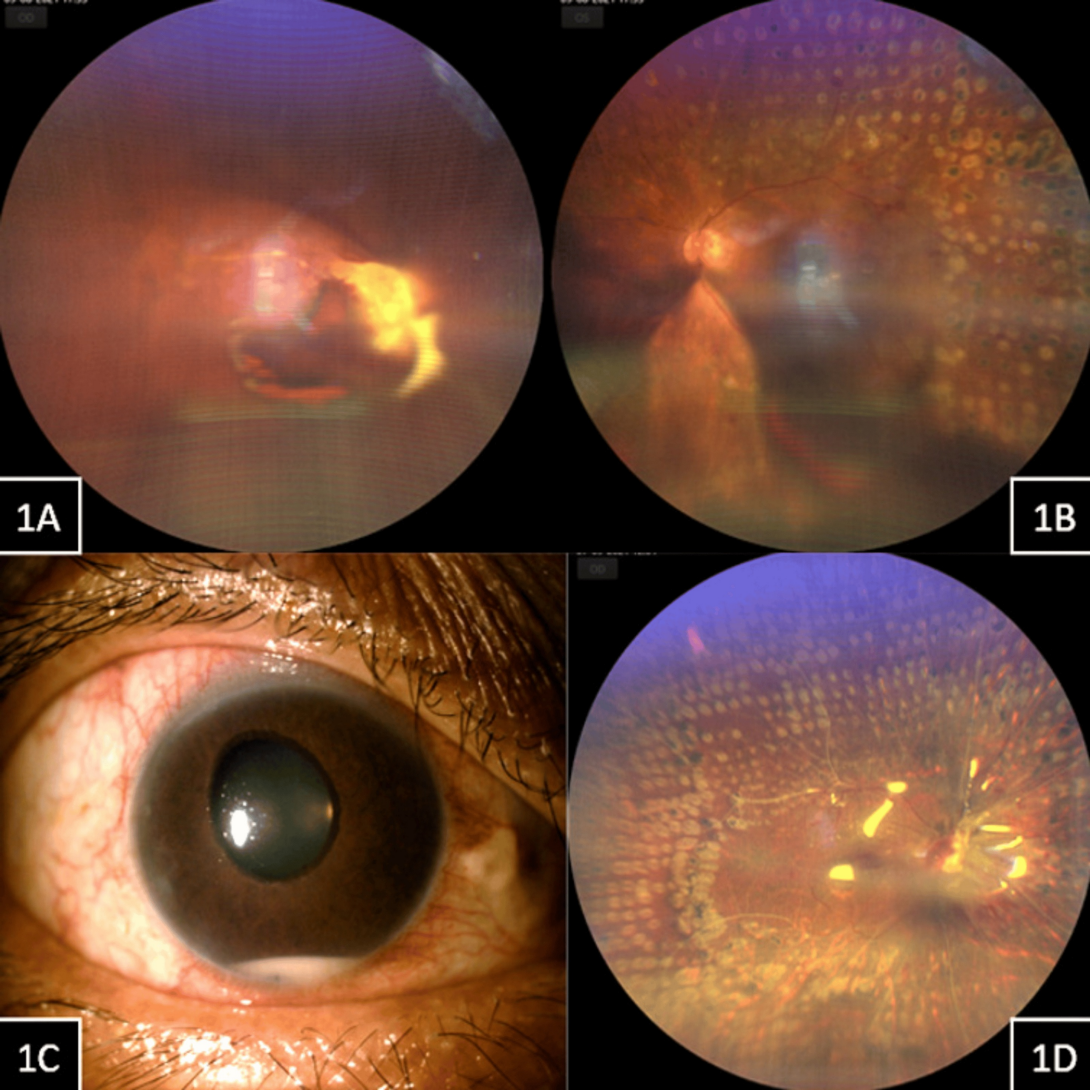
Case 1 Case 1, Figure 1A (Right eye) Figure 1B (Left eye): Right eye fundus showed the presence of proliferative diabetic retinopathy (PDR) with vitreous hemorrhage and sub hyaloid hemorrhage with fibrovascular proliferation at the disc. The left eye fundus showed the presence of PDR with inferior dispersed vitreous hemorrhage, posterior pole and superior half of the retina were visible with previous laser scars. Case 1, Figure 1C, 1D (Right eye): At 1- week post vitreoretinal surgery, the anterior segment of the right eye showed minimal conjunctival congestion, presence of 4+ cells with inferior mobile hypopyon in the anterior chamber and neovascularization of iris with ectropion uveae. Fundus examination of the right eye showed a pale disc with sclerosed vessels and attached retina under oil with laser scars

A 46-year-old man presented with complaints of bilateral painless progressive vision loss for one year. Systemic history was positive for type 2 diabetes mellitus, for which he was on insulin and oral hypoglycemics for the last two years. He also gave a history of bilateral pan-retinal photocoagulation done elsewhere for proliferative diabetic retinopathy a year ago. His best-corrected visual acuity at presentation was an appreciation of hand movements in the right eye and 20/320 in the left eye. The anterior segment evaluation of the right eye showed neovascularization of the iris with ectropion uveae and cataract with nuclear sclerosis of grade 2. Left eye anterior segment evaluation showed cataracts with nuclear sclerosis of grade 2. His intraocular pressures were 24 and 14 mmHg in the right and left eye, respectively. He was not on any anti-glaucoma medication. Dilated fundus evaluation in the right eye showed proliferative diabetic retinopathy (PDR) with vitreous hemorrhage and sub-hyaloid hemorrhage with fibrovascular proliferation at the disc (Figure 1A). The left eye fundus showed PDR with inferior dispersed vitreous hemorrhage, but the posterior pole and superior half of the retina were clearly visible with old laser scars (Figure 1B). A clinical diagnosis of right eye neovascular glaucoma and both eyes vitreous hemorrhage secondary to PDR was made. He was then planned for both eyes vitreoretinal surgery, the right eye followed by the left eye. He underwent pars plana vitrectomy with endo-laser, anterior retinal cryotherapy for anterior hyaloid proliferation, and silicone oil injection (1000 centistokes) in the right eye. On postoperative day 1, the vision in the right eye was an appreciation of hand movements, and intraocular pressure was 20mmHg. Anterior segment evaluation did not show any inflammation, and the fundus showed a pale disc with an attached retina under oil with laser scars. At one week post vitreoretinal surgery, the vision in his right eye was an appreciation of hand movements, and intraocular pressure was 26 mmHg. His anterior segment showed minimal conjunctival congestion, the presence of 4+ cells with inferior hypopyon in the anterior chamber (AC), and neovascularization of the iris with ectropion uveae (Figure 1C). No signs of lid edema or tenderness were noted in the right eye. The fundus examination of the right eye showed a pale disc with sclerosed vessels and an attached retina under oil with laser scars (Figure 1D).

No AC entry was made during the primary surgery, and none of the other patients who operated on the same day had a similar reaction to any of the intraocular solutions with the same batch numbers used during surgery. The patient was started on hourly topical steroid (1% prednisolone acetate), topical cycloplegic (2% homatropine hydrobromide) three times a day, and topical anti-glaucoma agent (0.5% timolol maleate) two times a day in the right eye and was called for review daily. The hypopyon completely disappeared within 48 hours of starting hourly topical steroids. A week later, the patient also underwent intracameral bevacizumab 1/3rd dose (0.4 mg/0.015 ml) in the right eye to reduce the anterior segment neovascularization. However, the visual prognosis was guarded in the right eye due to optic atrophy. A month later, the patient underwent left eye pars plana vitrectomy with endo-laser in view of non-clearing vitreous hemorrhage. He gained a good vision of 20/25 at one month postoperative review in the left eye with a healthy optic disc.

Case 2

A 52-year-old man presented with complaints of left eye painless progressive vision loss for six months. He had a history of type 2 diabetes mellitus and hypertension, for which he had been on oral medications for 13 years. He also gave a history of both eyes pan-retinal photocoagulation done elsewhere for proliferative diabetic retinopathy two years ago. His best-corrected visual acuity in the right eye was 20/40, and his left eye was counting fingers at 1 meter. The anterior segment evaluation in both eyes showed early cataracts with nuclear sclerosis of grade 1. His intraocular pressures were 16 and 14 mmHg in the right and the left eye, respectively. Right eye dilated fundus evaluation showed proliferative diabetic retinopathy (PDR) that was stable post pan-retinal photocoagulation (PRP). The left eye fundus showed dense sub-hyaloid hemorrhage due to unstable PDR. He was subsequently planned for left eye vitreoretinal surgery. He underwent pars plana vitrectomy with endo-laser and silicone oil injection (1000 centistokes) in the left eye. On postoperative day 1, the vision in the left eye was counting fingers at 2 meters, and intraocular pressure was 18mmHg. Anterior segment evaluation did not show any inflammation, and the fundus showed an attached retina under oil with laser scars.

**Figure 2:**
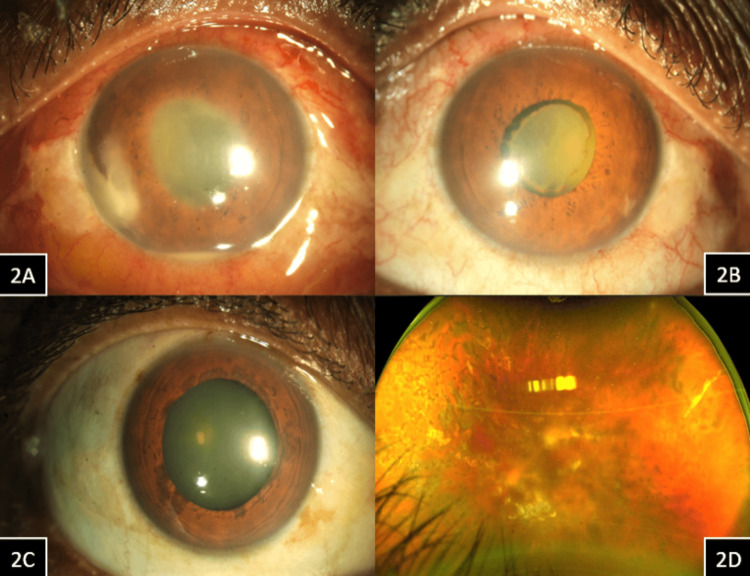
Case 2 Case 2, Figure 2A (Left eye): At one week post vitreoretinal surgery anterior segment showed conjunctival congestion, the presence of 4+ cells with inferior hypopyon in the anterior chamber, exudate plaque over the nasal iris surface and thin pupillary membrane. Case 2, Figure 2B (Left eye): After three days of starting hourly topical steroids, the inferior hypopyon had completely resolved, and the pupillary membrane was contracting, showing a favorable response to medical treatment. Case 2, Figure 2C (Left eye): After 10 days of starting frequent topical steroids, the pupillary membrane had completely resolved. Figure 2D (Left eye): The fundus showed an attached retina under silicone oil with post pan-retinal photocoagulation(PRP) laser scars.

At one week post vitreoretinal surgery, the vision in his left eye was counting fingers close to face, and intraocular pressure was 16 mmHg. His anterior segment showed conjunctival congestion, the presence of 4+ cells with inferior hypopyon in the anterior chamber, exudative plaque over the nasal iris surface, and a thin pupillary membrane (Figure 2A). The left eye fundus showed a dull glow with hazy retinal details. No AC entry was made during the primary surgery, and none of the other patients who operated on the same day had a similar reaction to any of the intraocular solutions with the same batch numbers used during surgery. The patient was admitted and was started on hourly topical steroids (1% prednisolone acetate) and topical cycloplegic (2% homatropine hydrobromide) three times a day. He was reviewed twice a day during hospital admission. On the third day of admission (i.e., postoperative day 10), the inferior hypopyon resolved completely, and the pupillary membrane was contracting (Figure 2B), showing a favorable response to treatment. The left eye fundus glow was better, and the retina was hazily seen. On the seventh day of admission, the pupillary membrane had completely resolved, and the patient was discharged with a tapering dose of steroid (1% prednisolone acetate) six times per day and topical cycloplegic (2% homatropine hydrobromide) three times a day. He was reviewed again a week later in the outpatient department, and the left eye anterior segment showed complete resolution of the pupillary membrane (Figure 2C). The left eye fundus showed an attached retina under silicone oil with PRP laser scars (Figure 2D). The vision in the left eye had improved to 20/200 during this visit.

Differential diagnosis

Toxic anterior segment syndrome (TASS) could be a differential considering the resolution of anterior chamber inflammation to frequent topical steroids. However, no AC entry was made during the primary surgery and none of the patients operated on the same day with the same batch number for the intraocular solutions, which developed similar complaints. Moreover, TASS presents within 48 hours of surgery.

Acute post-surgical endophthalmitis can be another close differential although unlikely in an oil filled eye post vitrectomy with no AC entry. Moreover, both the patients did not have lid edema or tenderness. Hospital admission or daily outpatient department review of the patient for resolution of hypopyon on frequent topical steroids without the cover of topical or systemic antibiotics is an efficient monitoring strategy to differentiate the two.

Outcome and follow-up

In case 1, the right eye vision did not improve because of optic atrophy and ischemic retina with sclerosed vessels. He was on topical anti-glaucoma medications in the right eye with an intraocular pressure of 20mmHg at the last visit. Left eye did well status post vitreoretinal surgery and remained stable at 2 months post-surgery. During his last follow-up, the visual acuity in the left eye was 20/25, and intraocular pressure was 14mmHg without any anti-glaucoma medication.

In Case 2, the left eye vision improved to 20/200 at 2nd week post-op. Subsequently, three months later, the patient underwent cataract surgery with silicone oil removal in the left eye. He is doing well post-surgery and is currently is following up in the local hospital. The right eye remained stable status after PRP.

## Discussion

Diabetic retinopathy is an inflammatory condition, and previous reports have shown that patients with uncontrolled blood sugars can develop diabetic anterior uveitis, which usually responds well to topical steroids and blood sugar control [5]. Hypopyon uveitis can also be expected post pan-retinal photocoagulation in patients with a previous history of anterior uveitis [6]. The extent of posterior segment ischemia, presence of neovascularization, longer surgical time, and use of cryotherapy during surgery could have been some of the factors that could have compromised the blood ocular barriers in our cases described above. Previous reports have also described impurities in silicone oil as one of the causes of acute inflammatory response post vitreoretinal surgery with silicone oil tamponade, but these were in non-diabetic retinopathy patients [7-9].

In both our cases, the patients were relatively asymptomatic without any lid edema or tenderness. Both the patients showed favorable responses to hourly steroids with daily close monitoring with slit lamp photography. This is a safe and effective strategy to prevent erring in the side of endophthalmitis and subjecting the patient to a surgical intervention or intraocular antibiotic injection. To the best of our knowledge, this is the first report on non-surgical management of inflammatory anterior chamber hypopyon post vitreoretinal surgery for PDR. 

## Conclusions

Inflammatory anterior chamber hypopyon post vitreoretinal surgery can be expected in proliferative diabetic retinopathy patients. Diabetic retinopathy is a condition with raised pro-inflammatory markers; these patients are more prone to the exaggerated and faster recruitment of inflammatory cells and mediators in an event like surgery where various blood ocular barriers could be compromised. The absence of lid edema or tenderness and favorable response to frequent topical steroids without topical or systemic antibiotic cover points towards inflammation rather than endophthalmitis. Hospital admission and daily monitoring for the resolution of hypopyon to hourly topical steroids is an efficient and safe strategy to differentiate between only inflammation and probable chances of endophthalmitis.

## References

[1] Quevedo-Martínez JU, Garfias Y, Jimenez J, Garcia O, Venegas D, Bautista de Lucio VM (2021). Pro-inflammatory cytokine profile is present in the serum of Mexican patients with different stages of diabetic retinopathy secondary to type 2 diabetes. BMJ Open Ophthalmol.

[2] Tsai T, Kuehn S, Tsiampalis N (2018). Anti-inflammatory cytokine and angiogenic factors levels in vitreous samples of diabetic retinopathy patients. PLoS One.

[3] Costagliola C, Romano V, De Tollis M (2013). TNF-alpha levels in tears: a novel biomarker to assess the degree of diabetic retinopathy. Mediators Inflamm.

[4] Shahulhameed S, Vishwakarma S, Chhablani J (2020). A Systematic Investigation on Complement Pathway Activation in Diabetic Retinopathy. Front Immunol.

[5] Watanabe T, Keino H, Nakayama K, Taki W, Echizen N, Okada AA (2019). Clinical features of patients with diabetic anterior uveitis. Br J Ophthalmol.

[6] Tyagi M, Ambiya V, Rani PK (2016). Hypopyon uveitis following panretinal photocoagulation. BMJ Case Rep.

[7] Pichi F, Hay S, Abboud EB (2020). Inner retinal toxicity due to silicone oil: a case series and review of the literature. Int Ophthalmol.

[8] Doctor MB, Parameswarappa DC, Rani PK (2021). Medical management of silicone oil associated acute postoperative ocular inflammation. BMJ Case Rep.

[9] Russo A, Morescalchi F, Donati S, Gambicorti E, Azzolini C, Costagliola C, Semeraro F (2018). Heavy and standard silicone oil: intraocular inflammation. Int Ophthalmol.

